# Intracavity Raman scattering couples soliton molecules with terahertz phonons

**DOI:** 10.1038/s41467-022-29649-y

**Published:** 2022-04-19

**Authors:** Alexandra Völkel, Luca Nimmesgern, Adam Mielnik-Pyszczorski, Timo Wirth, Georg Herink

**Affiliations:** 1 Experimental Physics VIII—Ultrafast Dynamics, University of Bayreuth, 95440 Bayreuth, Germany; 2 Theoretical Physics III, University of Bayreuth, 95440 Bayreuth, Germany; 3 Department of Theoretical Physics, Wrocław University of Science and Technology, 50-370 Wrocław, Poland

**Keywords:** Solitons, Raman spectroscopy, Lasers, LEDs and light sources, Nonlinear optics

## Abstract

Ultrafast atomic vibrations mediate heat transport, serve as fingerprints for chemical bonds and drive phase transitions in condensed matter systems. Light pulses shorter than the atomic oscillation period can not only probe, but even stimulate and control collective excitations. In general, such interactions are performed with free-propagating pulses. Here, we demonstrate intra-cavity excitation and time-domain sampling of coherent optical phonons inside an active laser oscillator. Employing real-time spectral interferometry, we reveal that Terahertz beats of Raman-active optical phonons are the origin of soliton bound-states – also termed “Soliton molecules” – and we resolve a coherent coupling mechanism of phonon and intra-cavity soliton motion. Concurring electronic and nuclear refractive nonlinearities generate distinct soliton trajectories and, effectively, enhance the time-domain Raman signal. We utilize the intrinsic soliton motion to automatically perform highspeed Raman spectroscopy of the intra-cavity crystal. Our results pinpoint the impact of Raman-induced soliton interactions in crystalline laser media and microresonators, and offer unique perspectives toward ultrafast nonlinear phononics by exploiting the coupling of atomic motion and solitons inside a cavity.

## Abstract (web summary)

Here, the authors demonstrate intra-cavity excitation and time-domain sampling of coherent optical phonons inside an active laser oscillator. They discover that Terahertz crystal vibrations link successive ultrashort solitons which offers an approach to highspeed Raman spectroscopy inside laser cavities.

## Introduction

**Fig. 1:**
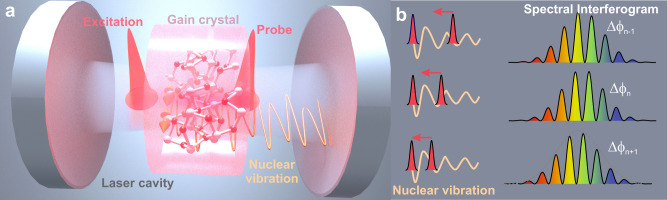
Intracavity Time-domain Raman Sampling. **a** Two ultrashort solitons (red) circulate inside an active laser cavity: The leading soliton excites a coherent nuclear vibration (orange) in the gain crystal, the trailing pulse samples the refractive index modulation. **b** During the soliton approach, the trailing soliton encodes the temporal waveform of the nuclear vibration in its phase (left). We detect the relative phases at each roundtrip $n$, $\triangle {\phi }_{n}$, via spectral fringes of single-shot interferograms (right).

Time-resolved sampling of coherent excitations comprises a key concept of pump-probe spectroscopy in experimental quantum physics and ultrafast sciences^1^. The probing of atomic motion on its natural timescale unveils phonon lifetimes, vibrational dephasing and couplings in condensed matter^2,3^. Particularly in transparent media, ultrashort pump pulses can impulsively stimulate coherent phonon motion via Raman scattering non-resonant with the electronic system. Impulsive stimulated Raman excitation allows for preparing, manipulating and controlling coherent phonon motion^4–6^—and may occur at every roundtrip inside an active ultrafast laser cavity, as we demonstrate in the following. Atomic lattice dynamics commonly remain hidden due to the fast phonon decay compared to the cavity roundtrip period of several nanoseconds. Here, we study the inter-soliton motion on femto- and picosecond timescales during laser operation based on two circulating solitons and unravel the link between impulsive stimulated Raman excitation and the largely unresolved interaction of multiple ultrashort pulses. In particular, we identify Raman-active optical phonons as the key drivers of soliton binding^7–9^ and complex inter-soliton trajectories^10^ inside a laser cavity, as sketched in Fig. 1a. Moreover, we demonstrate that real-time optical interferometry of soliton interactions serves as an unprecedented tool for coherent tracking of phonon dynamics and for stimulated Raman spectroscopy (SRS) in the temporal domain at very high speed^11–13^.

Recently, real-time spectroscopy based on the time-stretch dispersive Fourier transform (TS-DFT) has enabled high throughput measurements in laser science and nonlinear optics, yielding consecutive single-shot spectra exceeding 100 million frames per second. Following observations in passive nonlinear systems^14,15^, the scheme has been applied to nonlinear behavior inside ultrafast laser oscillators, opening up views into the buildup of soliton mode-locking and complex multi-soliton interactions in real-time^10,16–18^. Moreover, temporal solitons are observed to form stable and meta-stable bound states—termed “soliton molecules”—with non-commensurate temporal separation distances, spanning from picoseconds down to few 10-femtoseconds. Deterministic switching between such soliton molecules upon external control has also been demonstrated^19^. The temporal soliton separations in these systems match various earlier reports on double-pulsing in Kerr-lens modelocked Ti:sapphire lasers—the underlying mechanisms, however, have remained elusive^7–9^.

## Results

In this work, we analyze real-time measurements of transient multi-soliton dynamics in Kerr-lens modelocked lasers, based on experimental data and previous reports^8,10^. According to the common design of mode-locked Titanium-doped sapphire oscillators, the crystal serves as a broadband gain medium and as an ultrafast nonlinear amplitude modulator, enabling passive mode-locking via intensity-dependent focusing due to the spatial Kerr-lens effect^20,21^. Upon detuning of the resonator stability from single-pulse operation, mode-locking of two solitary pulses is readily obtained, which circulate principally independently inside the resonator. Yet, interactions based on the laser gain saturation and nonlinear refraction induce different group velocities and, thus, generate relative motion of both pulses^22^. As a result, the two solitons autonomously scan through their temporal separation and effectively perform degenerate pump-probe sampling in an all-optical and autonomous fashion. For large temporal separations of several hundred picoseconds, such relative motion is perceivable via fast real-time photodetection of the output intensity over many roundtrips.

**Fig. 2:**
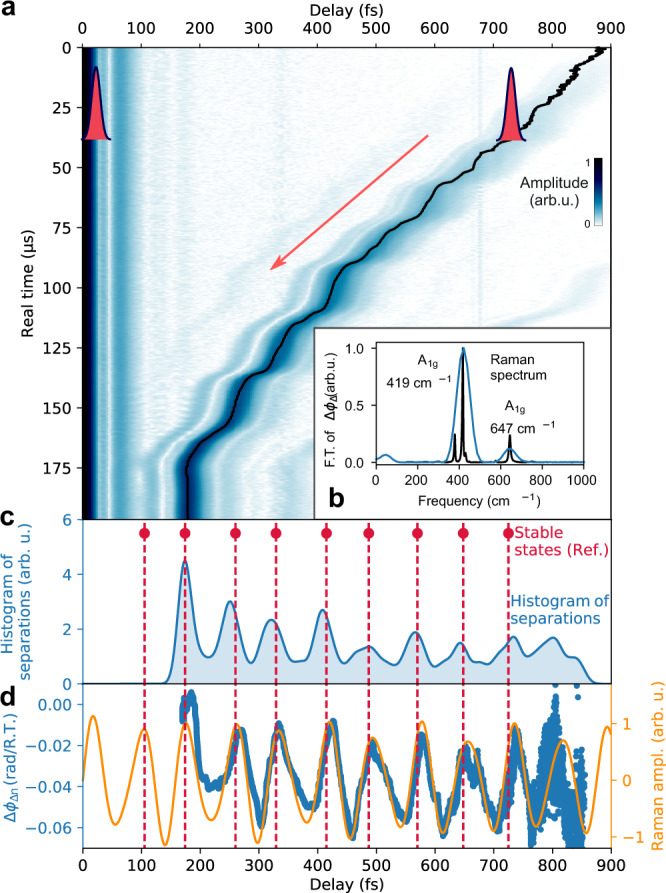
Transient approach of two solitons and extraction of the temporal Raman response. **a** The evolution of soliton separations is obtained via Fourier amplitudes of roundtrip-resolved interferograms, presenting field-autocorrelations. The side peaks represent the separation and are highlighted (black line). Experimental data from Ref. ^10^. **b** The resulting Raman spectrum obtained from Fourier transformation of the measured phase signal $\Delta {\phi }_{\Delta }\left(\tau \right)$ (see **d**) and a reference spectrum from the Raman database^26^. **c** The histogram of transient separations, derived as the sum of all autocorrelations, reveals the separations of meta-stable bound-states. The latter coincide with stable bound-states observed by Kitano et al. in Ref. ^8^, marked by red dots and dashed lines. **d** The relative phase-change per roundtrip, $\Delta {\phi }_{\Delta {{{{{\rm{n}}}}}}}\left(\tau \right)$, extracted via consecutive double-referencing, yields the temporal Raman waveform. For comparison, the theoretical Raman response is included in orange, and evidences that bound-states form at local maxima, i.e., phonon beats.

The critical dynamics on pico- to femtosecond separations are not resolved via direct photodetection, but can be tracked on a continuous, single-shot basis employing TS-DFT and spectral interference. We track the inter-soliton motion via recording single-shot spectra that encode soliton separations and relative phases via spectral interference, as illustrated in Fig. 1b. Experimental real-time data of a soliton approach from 900 to 180 fs separation are presented in Fig. 2a:^10^ At each roundtrip, we map the Fourier amplitudes of the respective spectral interferograms that represent field-autocorrelations. Thus, the side peak of the symmetric dataset reveals the relative soliton separation (mirror-symmetric negative delays omitted). We observe that both solitons approach via steps at separations in the picosecond to 100 fs range. Such meta-stable separations appear incommensurably spaced and exhibit varying degrees of stability. In order to analyze these dynamics, we generate a histogram of dynamic separations by integrating the roundtrip-resolved autocorrelation peaks along consecutive traces on the real-time axis. The result is shown in Fig. 2c and displays distinct maxima. Notably, we observe that all pronounced separations essentially coincidence with stable bound-states reported in early studies of Kerr-lens mode-locking in Ti:sapphire, as marked by red circles and dashed lines in Fig. 2c, including the final state at 180 fs.

Given these observations, we can directly unravel the underlying origin of the observed soliton molecule motion by relating the data to the temporal Raman response of the laser host medium, shown in Fig. 2d (orange line). The titanium-doped host crystal of the active laser medium is sapphire (Al_2_O_3_) and corresponds to the crystal structure of the rhombohedral space group R-3c. The birefringent crystal is cut at Brewster’s angle and is aligned with the optical axis parallel to the linear polarization of the beam inside the crystal. Referring to Porto’s notation, the Raman selection rules for the corresponding *X(ZZ)X*-geometry allow for accessing two *A*_1g_ modes given by optical Terahertz phonons at frequencies of 645 1/cm and 418 1/cm or 16 THz and 13 THz, respectively^23,24^. Following the impulsive Raman excitation by the first soliton at delay $\tau =0$, the two coherent modes generate an exponentially decaying waveform, and we find that the resultant local maxima of the phonon beats govern the bound-state separations. The physical mechanisms are discussed in further detail below.

Moreover, we now demonstrate the time-domain Raman spectroscopy of phonon vibrations based on this single trajectory of two approaching solitons, captured within an acquisition time below 170 µs. The scheme is enabled by introducing “consecutive double-referencing” for sensing delay-dependent relative phase changes: Whereas the Fourier amplitudes of the spectral interferograms yield the field-autocorrelations, the fringe positions encode the relative phase ∆*ϕ*(*τ*) between two pulses with fields ${E}_{{{{{\mathrm{1,2}}}}}}$, temporally separated by $\tau$ [Eq. 1]:

1 $${|E(\omega )|}^{2}= 	\,\frac{1}{2}\{{|{E}_{1}(\omega )|}^{2}+{|{E}_{2}(\omega )|}^{2}\}+|{E}_{1}(\omega )|\cdot |{E}_{2}(\omega )|\\ 	\cdot \,\cos ({\omega }_{0}\tau +\varDelta \phi (\tau )).$$

Evaluated at roundtrip *n*, it yields the relative phase $\triangle {\phi }_{n}(\tau )$ of a bound-state at separation $\tau$. In contrast, the double-referenced difference signal tracks the change of the relative phase to the next cavity roundtrip, $\triangle {\phi }_{\triangle n}\left(\tau \right)=\triangle {\phi }_{n}-\triangle {\phi }_{n-1}$, sketched in Fig. 1b. This observable is sensitive to round-trip differences in the effective index of refraction arising from the delayed temporal Raman response, sampled at inter-soliton separations τ. We evaluate this double-referenced phase signal as a function of pump-probe delay τ and we find that the observable tracks the actual Raman response of the medium with high fidelity, as demonstrated in Fig. 2d (blue) and evidenced by the theoretical Raman response derived from conventional Raman data^23^ (orange). The time-domain data yield complex-valued Raman spectra via Fourier transformation^25^ and the resultant Raman spectrum is presented in Fig. 2b, along with a reference spectrum from spontaneous Raman scattering^26^ (black, RRUFFID=R110119 at different geometry). The quality of the Raman signal depends on cavity alignment, as the intrinsically nonlinear laser behavior allows for diverse sets of trajectories at different operation parameters. Yet, characteristic Raman signatures are found in every dual-soliton trajectory, whereas laser instabilities and noise may induce deviations of sampled Raman waveforms (see Supplementary Information).

**Fig. 3:**
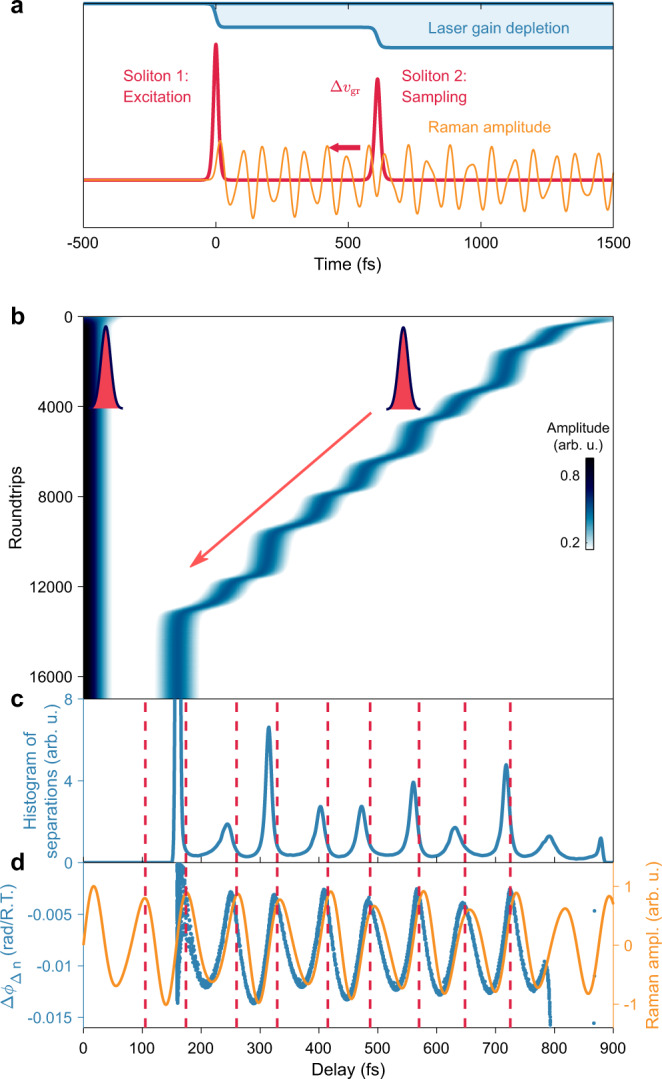
Simulation of soliton dynamics. **a** Intracavity Raman sampling is based on the relative motion of two solitons (red) inside an ultrafast laser provided by group velocity differences $\Delta {v}_{{gr}}$ due to gain depletion (blue) and self-steepening. Soliton 1 excites coherent lattice vibrations (orange) via impulsive simulated Raman scattering. The trailing soliton 2 samples the ultrafast refractive modulation—arising from combined nonlinear electronic and nuclear contributions in the Kerr-lens configuration. **b** Simulated evolution of single-shot autocorrelations as a function of cavity roundtrips during a soliton approach. In correspondence to the experimental observations, the motion progresses via meta-stable steps and finally stops at a stable bound-state. **c** The histogram of separations during the transit reveals meta-stable states, close to stable bound-state separations observed by Kitano et al. (red, Ref. ^16^). **d** The extracted relative phase differences between two consecutive simulation roundtrips, $\Delta {\phi }_{\Delta {{{{{\rm{n}}}}}}}\left(\tau \right)$, is governed by the underlying Raman waveform (orange).

We now discuss the coupling mechanism based on the analysis of a nonlinear numerical propagation model^27^ and supporting experimental data (see Supplementary Information). The underlying soliton interaction evolves along the process sketched in Fig. 3a: Two initially separated solitons (red) experience different laser gain due to transient gain depletion (blue). Whereas closely spaced pulses in modelocked lasers without Kerr-lensing typically separate further apart due to the dominant role of the gain gradient^18,28^, here, the high Kerr nonlinearity is accompanied by significant self-steepening. In line with experimental observations, the resultant intensity-dependent group velocity difference $\Delta {v}_{{gr}}$ slows the first soliton and induces approaching relative motion. Particularly, each soliton impulsively stimulates the allowed lattice vibrations that are accessible in the Raman-active medium. The nuclear motion (orange) excited by the leading pulse contributes a nuclear component to the nonlinear refractive index modulation $\triangle n$ at delay $\tau$, $\triangle n\left(t-\tau \right)={n}_{2,{el}}I\left(t-\tau \right)+{\int }_{0}^{\infty }R\left(u\right)I\left(t-\tau -u\right){du}$, with the temporal response function $R\left(u\right)$. In the impulsive limit, the delay-dependent nuclear index modulation experienced by the trailing pulse yields: $\triangle {n}_{2,{nuc}}\left(\tau \right)=R\left(\tau \right)\cdot I$. The effective Raman response for the sapphire laser crystal after impulsive stimulation corresponds to a free exponential decay of $n=2$ dominant optical *A*_1g_ phonon modes: $R\left(\tau \right)={\sum }_{i=1}^{n}{R}_{n}\cdot {{{{{\rm{exp }}}}}}(-\tau /{T}_{{R}_{n}})\cdot {{{{{\rm{sin }}}}}}(2\pi {\Omega }_{n}\tau)$ at frequencies ${\Omega }_{1}=16$ *THz* and ${\Omega }_{2}=13$ *THz*, respectively. Measurements of this time-delayed subtle index contribution typically utilize the transformation to intensity modulations, e.g., employing polarization rotation, spectral shifts or Raman-induced Kerr-lensing – in combination with lock-in amplification and averaging^3,29^.

Inside the Kerr-lens mode-locked cavity, a transformation of nonlinear phase into amplitude modulation is intrinsically provided^20^. As a result, the time-dependent Raman-induced amplitude modulation of the trailing soliton changes the group velocity difference upon relative soliton motion. In fact, the data in Fig. 2c, d reveal that the motion decelerates and meta-stable binding occurs at the local maxima of the Raman waveform. Based on the intricate balance of gain saturation, electronic and Raman nonlinearities, our model reproduces this characteristic experimental observation, as shown in the simulated autocorrelations and derived histogram in Fig. 3b, c, respectively. We find similar agreement only by including both Raman-induced phase and amplitude modulation, and we employ relative strengths according to the ratio of electronic self-phase and self-amplitude modulation in Ti:sapphire lasers^30^ of 10. In correspondence to the experiments, the motion may not necessarily stop, and upon continued relative motion, the nuclear index modulation changes its sign. At this point, the relative soliton motion reaccelerates again, and effectively, generating an overall stepwise trajectory. As the phonon amplitudes increase toward smaller delays, eventually, the increased Raman-interaction counterbalances the group velocity difference and stable bound-states form. In the model, we also reproduce the observed final stable binding, however, we note that this evolution represents a particularly prominent and exemplary trajectory, and different and more complex inter-soliton behavior is also obtained in simulations and experiments upon changes of conditions and parameters (see more examples in the Supplementary Information).

Following on insight into the soliton trajectory, we focus onto the apparent high signal quality of the Raman waveform extracted from the double-referenced phase $\triangle {\phi }_{\triangle n}\left(\tau \right)$, as it facilitates fast Raman sampling via highspeed real-time spectroscopy. Inside the cavity, an advantageous combination of important requirements for time-domain Raman spectroscopy is given: First, intra-cavity power enhancement and dispersion compensation generate intense and ultrashort pump/probe pulses inside the sample. Second, the introduced difference signal $\triangle {\phi }_{\triangle n}\left(\tau \right)$ from two successive roundtrips effectively suppresses critical low-frequency noise contributions via fast consecutive referencing. Third, our signal generation inside the Kerr-lens modelocking geometry is considered to enhance the Raman signal via the interlinking of Raman-induced amplitude modulation and electronic self-phase modulation. In contrast to SRS implementations based on Kerr-lensing outside the cavity^31–33^, here, Raman information is not obtained from amplitude modulations but phase changes encoded into spectral interferograms. Based on extra-cavity spectrally resolved two-beam coupling (SRTBC) measurements, we obtain an upper limit of the intrinsic nuclear versus electronic contribution to ${n}_{2}$ below <1%. Compared to typical electronic nonlinear phase shifts of ~1 rad per roundtrip inside Ti:Sapphire lasers^30^, the measured intra-cavity Raman phase oscillation of ~0.05 rad yields signal enhancements exceeding a factor of five (see Supplementary Information). Our numerical analysis indicates that both phase- and amplitude modulations driven in-phase with the Raman waveform are required to reproduce various features of the experimental trajectories and Raman-induced phase modulations, as shown in Fig. 3c, d. In particular, we observe that direct Raman-induced phase modulation alone or pure amplitude modulations do not yield qualitatively comparable evolutions of binding separations and relative-phase waveforms. Finally, we note that this advantageous soliton coupling is not limited to phonon modes in laser gain media only: Rapid intra-cavity Raman sampling of non-laser media, such as transparent solids or liquids, is also possible by employing the sample-induced Raman-lensing in an additional cavity focus together with the spatially-separated amplitude transformation given by the Kerr-lens modelocking mechamism.

## Discussion

**Fig. 4:**
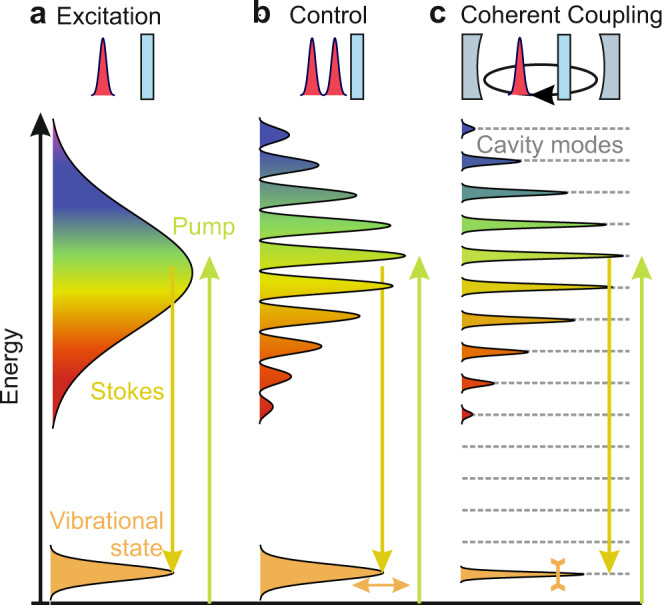
From excitation to coherent coupling of phonons to phononic wave-packets. **a** Optical quanta of a single pulse impulsively stimulate a vibrational mode via suitable difference frequencies. **b** Coherent control of the phonon excitation via a pair of ultrashort solitons. **c** Inside a short cavity, the mode-spacing of the resonator is matched to the vibrational phonon mode, allowing for feedback and coherent coupling of soliton wave-packets to phonon motion. Resonant pumping induces, e.g., the narrowing of vibrational linewidths.

In our experimental configuration, the cavity roundtrip duration exceeds the phonon lifetimes, yet, the general coupling mechanism can directly be applied to long-lived optical phonons and miniaturized cavities. Thus, the results may extend the interactions found with low-frequency acoustic phonons to high-frequency optical phonons^34–37^. Active crystalline microresonators offer further degrees to tailor and strengthen soliton-to-phonon coupling^38,39^. Fundamentally, beyond the well-established phonon excitation via a single soliton and the coherent control of phonon dynamics via tailored pulse pairs^4,40–42^ (Fig. 4a, b), the optical feedback inside short active cavities with optical mode spacings matched to phonon modes may further enable resonant phonon pumping, stabilization of Terahertz pulse trains or “soliton crystals”^43^, and eventually allow for ultrafast hybrid photonic-phononic devices and phonon-lasing^44,45^, illustrated in Fig. 4c.

In conclusion, we reveal impulsive stimulated Raman scattering of optical phonons as a fundamental driving force for the coupling of ultrafast temporal solitons inside cavities with crystalline active media. The process is mediated via the Kerr nonlinearity in temporal and spatial domains, and manifests upon inter-soliton motion. As a result, the impulsively stimulated lattice motion generates stable and meta-stable soliton bound-states at Terahertz phonon beats, and more complex trajectories are accessible via cavity tuning. Our results might enable the exploration of transient soliton dynamics for rapid and autonomous time-domain Raman spectroscopy. Furthermore, the underlying coupling mechanism between temporal solitons and phonons offers unprecedented routes to ultrafast cavity phononics^46,47^.

## Methods

The experiments are based on a commercial 20 fs Kerr-lens mode-locked Ti:sapphire laser oscillator. High-speed soliton detection is implemented via two channels of a real-time oscilloscope with 40 GSa/s sampling rate and 8 GHz analog bandwidth, using direct detection by a fast photodiode and spectral interferometry via the time-stretch dispersive Fourier transformation (TS-DFT), respectively. Spectra are dispersed using 600 m length of single mode optical fiber, enabling the resolution of soliton separations up to 2000 fs. Extra-cavity reference measurements are performed via SRTBC and Kerr-lens sampling with lockin-detection. Additional data, details on data analysis and simulation are provided in the Supplementary Information.

## Supplementary information

Supplementary Information

## Data Availability

The data that support the manuscript and other findings of this study are available from the corresponding author upon reasonable request.

## Code availability

The data processing and simulation codes to generate the figures in the paper and other findings of this study are available from the corresponding author upon reasonable request.
