# Supplementary material for: Intracavity Raman scattering couples soliton molecules with terahertz phonons
Source: Nat Commun. 2022 Apr 19;13:2066. doi: 10.1038/s41467-022-29649-y (PMC9018723; doi:10.1038/s41467-022-29649-y)
Supplement: Supplementary file 1 — Supplementary Information [file 41467_2022_29649_MOESM1_ESM.pdf]

Supplementary Information

Intracavity Raman Scattering couples Soliton Molecules with Terahertz Phonons

Alexandra Völkel[1], Luca Nimmesgern[2], Adam Mielnik-Pyszczorski[2,3], Timo Wirth[1], and Georg Herink[1*]

[1] Experimental Physics VIII – Ultrafast Dynamics, University of Bayreuth, 95440 Bayreuth, Germany
[2] Theoretical Physics III, University of Bayreuth, 95440 Bayreuth, Germany
[3] Department of Theoretical Physics, Wrocław University of Science and Technology, 50-370, Wrocław, Poland
Correspondence to: georg.herink@uni-bayreuth.de

1) Supplementary Real-Time Data

In the main article, we demonstrate that inter-soliton trajectories sample Raman-induced phonon vibrations with high fidelity under suitable laser operation conditions. Generally, a broad variety of highly complex soliton trajectories is obtained in modelocked sources, arising from a large set of concurring mechanisms - forming the basis of intense research activities in the field of ultrafast laser physics. The interaction is tunable e.g. via pump power and cavity alignment [1,2] and may also result in periodic and aperiodic trajectories. In this study, we report that the temporal Raman response in Ti:sapphire lasers still manifests essentially in all measurements of inter-soliton trajectories. In order to illustrate the diversity of Raman-affected soliton trajectories, we present additional exemplary experimental real-time data which are all depending on the Raman-induced soliton coupling, yet, to varying degrees depending on fine-tuning of the interaction.

Figure S1a presents a particularly fast soliton approach with high group velocity differences. The field-autocorrelations are obtained from the Fourier transformation of experimental realtime interferograms and the soliton separation is given by the Fourier amplitude, i.e. the satellite peak of the field autocorrelations. The inset presents the Fourier spectrum - deduced Raman spectrum - that we obtain from the Fourier transformation of the relative phase signal $\Delta\phi_{\Delta}(\tau)$, as elaborated in the main article for Fig. 2d. The spectrum is dominated by the low-frequency optical phonon mode at the frequency of 419 1/cm. The absence of the second, high-frequency mode indicates that the pulse duration is broadened in this operation mode. As a result, both high-frequency phonon excitation and time-domain sampling are not effective for high-frequency phonon oscillations – illustrating the basic requirement for time-domain sampling schemes.

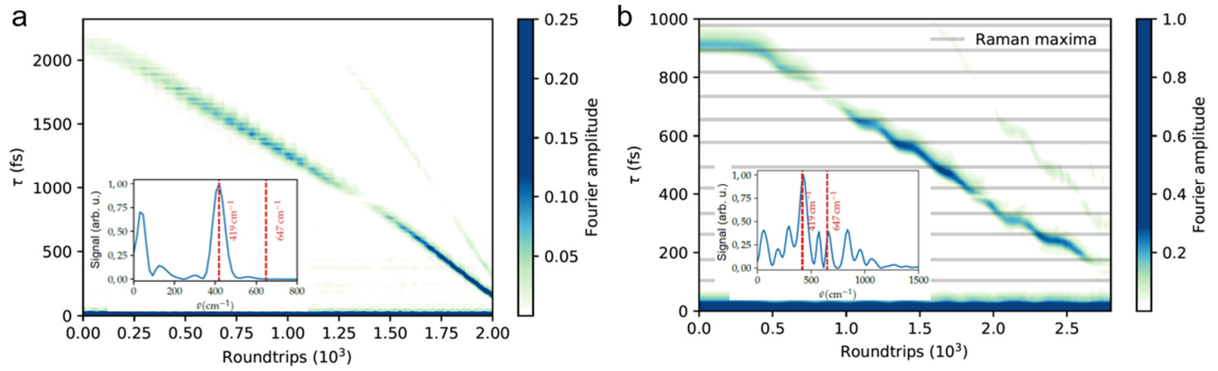

Fig. S1: Rapidly approaching and meta-stable solitons: a) Two solitons continuously approach at high speed, as shown by the evolution of field-autocorrelations, obtained from single-shot interferograms. The inset shows the Raman spectrum that is encoded in the evolution of the relative soliton phase. b) Soliton approach via meta-stable states and corresponding Raman spectrum. The maxima of the theoretical Raman waveform are indicated by grey lines.

Next, we display a soliton approach via meta-stable separations in Fig. S1b, similar to the data of Fig. 2 in the main text. In this measurement, the laser was perturbed via translation of an intra-cavity prism, leading to additional intensity fluctuations. We observe that solitons transiently persist at all maxima of the Raman response, indicated by the grey horizontal lines. The deduced Raman spectrum reveals the dominant oscillation of the low-frequency phonon mode. The high frequency mode appears, yet, the spectrum displays various structures of similar strength which are not identified and which we assign to artefacts from intensity fluctuations in this less stable operation mode.

Finally, we present two limiting scenarios of soliton trajectories that illustrate the impact of the phonon amplitudes onto soliton motion, however, the evolutions of the relative phases are not dominating by the effect of Raman-induced phase shifts. In Fig. S2a, two solitons approach via metastable separations, however, not all Raman beating maxima are sufficient effective to transiently bind both solitons.
In Fig. S2b, two solitons continuously approach and separate. The turning points of the motion are given by maxima of the Raman waveform indicated by grey lines.

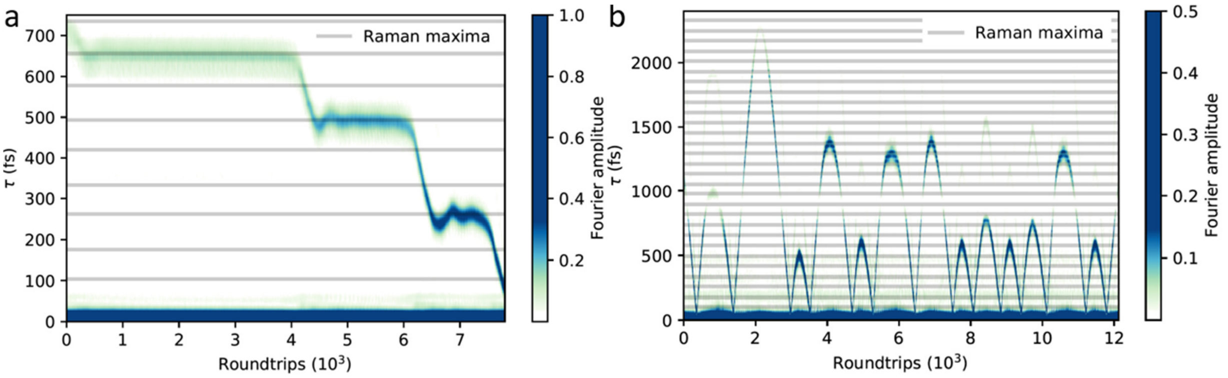

Fig. S2: a) Meta-stable soliton separations: Two solitons approach via metastable separations, given by selected maxima of the Raman waveform (maximum amplitudes indicated by grey lines). **b)** Repelling and approaching solitons with turning points at maxima of the Raman waveform indicated by grey lines.

2) Experimental methods and data analysis via consecutive double-referencing

The experiments are based on a commercial Kerr-lens mode-locked Ti:sapphire 20-fs laser oscillator with prism-based dispersion compensation following the prototypical implementation of Sibbet with soft-aperture Kerr-lens modelocking. The operating principle is identical to systems employed in Refs. [1-3]. Minor differences arise from the degree of dispersion compensation via double-chirped mirrors in the latter sub-10fs system, and the power levels of pump and output. The consistency of our results with earlier observations corroborate the universal applicability of our findings to similar designs based on Ti:sapphire gain and Kerr media.

High-speed temporal soliton detection is implemented with two channels of a real-time oscilloscope with 40 GSa/s sampling rate and 8 GHz analog bandwidth. Upon splitting the oscillator output, one beam is directly detected by a fast photodiode, yielding 150 ps temporal resolution for tracking large soliton separations. The second beam is employed for the detection of pico- to femtosecond pulse separations via spectral interferometry. The spectra are effectively mapped to the temporal domain via the time-stretch dispersive Fourier transformation (TS-DFT), realized via dispersing the pulses with 600 m length of single mode optical fiber and total group velocity dispersion of $155{,}0\,\mathrm{ps/nm}\cdot\mathrm{km}$ at a central wavelength of 770 nm. The scheme allows for resolving soliton separations up to 2000 fs.

Consecutive temporal waveforms represent roundtrip-resolved temporal and spectral information and are typically acquired over a total acquisition time of 2 ms, given by the memory depth of the oscilloscope. The traces are segmented with respect to the roundtrip duration and the spectra are calibrated to frequency or wavelength axis using the dispersion profile of the fiber and static reference spectra.
The introduced interferometric phase analysis is based on “consecutive double-referencing”: The calibrated spectral interferograms encode soliton separation and relative phase, according to the Fourier relations. The electric fields of two cavity solitons with additional phase $\varphi_{1,2}$ are given by:

$$E_{1,2}(t) = \left|E_{1,2}(t)\right| e^{i\varphi_{1,2}(t)}$$

The resultant spectral interferogram encodes the relative spectral phase differences $\Delta\phi(\omega)$ of the pulses[8]:

$$|E(\omega)|^2 = \frac{1}{2}\{|E_1(\omega)|^2 + |E_2(\omega)|^2\} + |E_1(\omega)| \cdot |E_2(\omega)| \cdot cos\big(\omega_0\tau + \Delta\phi(\omega)\big)$$

Evaluated at the maximum of the Fourier transform, we obtain the relative phase difference $\Delta\phi$ within the soliton pair. This observable is sensitive to accumulated phase variations, e.g., due to prevailing intensity differences and mediated by self-phase modulation. Referencing the relative phase difference $\Delta\phi_{n-1}$ of one roundtrip $n-1$ to the next consecutive roundtrip n, $\Delta\phi_n$, yields the consecutive double-referenced signal $\Delta\phi_{\Delta n} = \Delta\phi_n - \Delta\phi_{n-1}$. We find that this signal balances slow erratic overall phase fluctuations. Whereas the total relative phase varies at a fixed temporal separations τ, the consecutive double-referenced signal yields a stable and highly sensitive observable $\Delta\phi_{\Delta n}(\tau)$ to access delay-dependent modulations due to the Raman effect.

3) Extra-cavity reference measurements for the Raman-induced nonlinearity

In order to provide an estimate on Raman signal strengths in the introduced intra-cavity time-domain Raman sampling scheme, we performed reference measurements based on conventional time-domain Raman spectroscopy outside the laser cavity. Different measurement techniques may yield different relative contributions. We implemented spectrally resolved two-beam coupling measurements (SRTBC, based on the detection of a spectrally filtered fraction of the transmitted probe beam) and Kerr-lensing sampling spectroscopy (based on detection of a spatially cut fraction of the transmitted probe beam) with sapphire and Ti: sapphire crystals. Employing the output of a Ti:sapphire oscillator, we pump the sample with sub-40 fs pulses at few nJ pump energy, focused via an achromatic lens with a focal length of 40 mm. The probe signals are lockin-amplified and averaged over several tens of pump-probe time-traces.

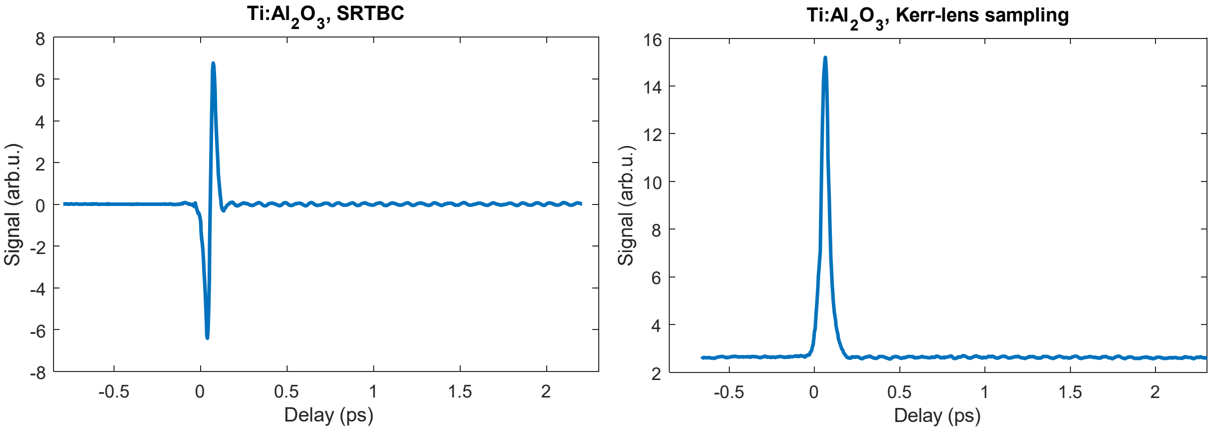

Fig. S3: Spectrally-resolved two-beam-coupling (SRTBC) and Kerr-lens Raman sampling of a Ti:sapphire laser crystal, displaying the contributions of electronic and nuclear nonlinear responses in each measurement scheme. Following the electronic nonlinearity, a significantly weaker nuclear waveform is apparent which arises from the two optical phonon modes. The orientation of the crystal is identical to the orientation for laser operation.

First, we performed a reference measurement via SRTBC of a Ti:sapphire crystal with identical orientation as used inside the laser. The time-trace is shown in Fig. S3 (left) and resolves the dominant initial electronic contribution and the time-delayed oscillating nuclear contribution. We obtain a relative amplitude fraction of nuclear to electronic nonlinear contributions of 0,88(11)%, in agreement with reference data from Smolorz et al. [Ref.4].
Next, we applied time-resolved Kerr-lens sampling, displayed in Fig. S3 (right). This scheme tends to yield a slightly lower relative fraction of 0,79(16)%.
In comparison, the real-time measurements via soliton motion display a typical nuclear peak-to-peak signal amplitude of the observable $\Delta\phi_{\Delta n} = 0.05\ rad$. With respect to typical roundtrip values of the purely electronic self-phase modulation of ~1 rad [5], the nuclear contribution amounts to 5%. Given to the above estimation of the relative nuclear signal below 1%, we estimate signal enhancements of the intracavity scheme exceeding a factor of 5.

4) Simulations

The dynamics arising from the interplay of soliton evolution inside the active cavity and Raman excitations of the crystal are studied using a master equation model based on an adapted complex Ginzburg-Landau Equation CGLE [6]

$$\frac{\partial}{\partial z}A(z,t) = \left[\alpha(t) - \rho - \frac{\Gamma_{\mathrm{sat}}}{1+\sigma_{\mathrm{sat}}|A(z,t)|^2} + i\gamma_0|A(z,t)|^2\right]A(z,t) + \left(t_f^2 + i\frac{\beta_2}{2}\right)\frac{\partial^2}{\partial t^2}A(z,t) - \gamma_1\frac{\partial}{\partial t}\left(|A(z,t)|^2A(z,t)\right) + iA(z,t)\,(R_p + iR_a)\int_{-\infty}^{t}\mathrm{d}\tau\, h_R(t-\tau)\,|A(z,\tau)|^2.$$

We numerically propagate the dissipative solitons via the slowly varying field envelope $A(t)$ in the presence of laser gain α, output coupling loss ρ, self-phase modulation γ_0 and group velocity dispersion β_2. The effective saturable absorption due to electronic Kerr-lensing is included via the modulation depth Γ_{sat} and saturation parameter σ_{sat}. The spectrally confined laser gain is characterized via the inverse fluorescence bandwidth t_f.

Moreover, we identified additional elements that are critical to our dynamics: Transient laser gain depletion in the absence of pump recovery on short timescales is approximated via the cumulative gain depletion

$$\alpha(t) = \alpha_0\left(1 - \kappa\int_{-\infty}^{t}\mathrm{d}\tau|A(z,\tau)|^2\right),$$

where κ is the depletion coefficient and α_0 is the linear gain. We also include temporal asymmetry via the intensity-dependence of the group velocity of ultrashort pulses due to self-steepening, introduced by the derivative term proportional to $\gamma_1 = \gamma_0/\omega_0$ and arising from higher order nonlinear self-phase modulation. In combination with the gain depletion it generates a difference of group velocities of the two solitons.

In order to include the refractive index perturbations by the Raman response, the corresponding temporal waveform is evaluated by convoluting the field intensity $|A(t)|^2$ with the linear response function

$$h_R(t) = R_1\exp\left(-\frac{t}{\tau_1}\right)\sin(2\pi\,\Omega_1\,t) + R_2\exp\left(-\frac{t}{\tau_2}\right)\sin(2\pi\,\Omega_2\,t),$$

describing phonon excitation in the impulsive limit. The effective Raman response of our gain medium is governed by two dominant phonon modes at $\Omega_1 = 16$ THz and $\Omega_2 = 13$ THz, respectively, with lifetimes $\tau_1 = 2$ ps and $\tau_2 = 6$ ps. We use relative amplitudes $R_1 = 0.5$ and $R_2 = 1$ in agreement to extra-cavity pump-probe measurements [4] and to our experimental data. In particular, the Kerr-lensing geometry induces an intensity-dependent amplitude modulation, adjustable via cavity alignment [5,7,8]. We estimate the relative contributions of the Raman-induced self-phase modulation R_p and self-amplitude modulation R_a to the total delayed nuclear response via the correspondence to the purely electronic self-phase and self-amplitude modulation in Ti:sapphire lasers, determined to be |SPM/SAM| ≥ 10 [5,9].
The experimentally observed coupling is obtained for a modulation strength of $(10 - i) \times 2.5 \times 10^{-6}$ m/Ws.

The pulse envelope is propagated through the crystal over small discretization segments with the Split-Step Fourier Method and a single roundtrip is defined as a distance equal to double crystal length x. A soliton in the fs-regime exhibits high peak power implying short non-linear length $L_{NL} = 1/(\gamma_0 P_0)$ and also prevents usage of the first Raman moment

approximation. To reduce huge overall computational cost we account for the Raman response using fast Fourier transform within the convolution theorem, namely

$$R(t) = \mathcal{F}^{-1}\{\mathcal{F}[h_R(t)] \cdot \mathcal{F}[|A(t)|^2]\},$$

where · denotes point-wise multiplication. Simulation parameters are provided in Table S1.

In order to illustrate the impact of individual perturbations to the inter-soliton motion, we present the effect of key terms onto the inter-soliton trajectory in Fig. S4.

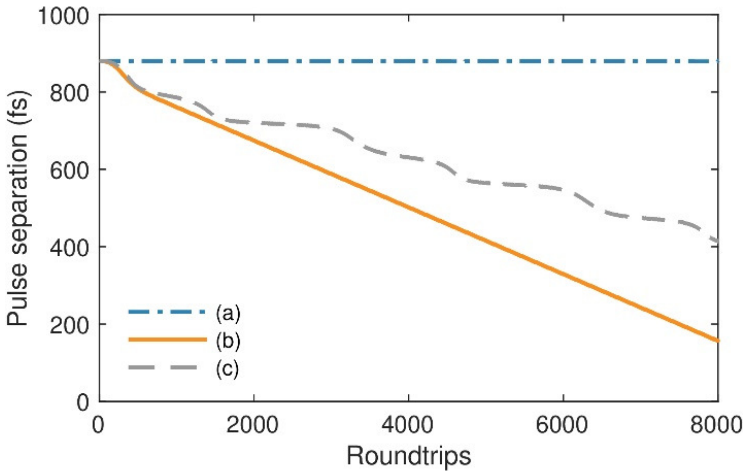

Fig. S4: Inter-soliton dynamics obtained by selectively adding key propagation terms to the model: (a) $R = 0, \gamma_1 = 0$, (b) $R = 0$, and (c) full model.

Considering the effect of gain depletion, a trailing soliton following a leading soliton experiences slightly lower gain, however, the group velocities are essentially similar, as shown Fig. S4 via constant inter-soliton separation (blue).

The interplay of intensity-dependent group velocity introduced by self-steepening results in a lower group velocity of the leading pulse and, thus, induce the continuous approach (orange). The full model shows characteristic meta-stable steps in the separation (grey).

Parameter	Value
x	6 mm
α_0	$20.8\ \mathrm{m}^{-1}$
ρ	$0.05\ x^{-1}$
γ_0	$1.34 \times 10^{-13}\ \mathrm{m\ W}^{-1}$
γ_1	$5.69 \times 10^{-29}\ \mathrm{m\ s\ W}^{-1}$
Γ_{sat}	$0.075\ x^{-1}$
σ_{sat}	$2.41 \times 10^{-17}\ \mathrm{m}^2\ \mathrm{W}^{-1}$
β_2	$3 \times 10^{-25}\ \mathrm{s}^2/\mathrm{m}$
t_f	$2.5 \times 10^{-15}\ \mathrm{s}\ x^{-1/2}$
κ	$3.5 \times 10^{-6}\ \mathrm{m}^2\ \mathrm{W}^{-1}\ \mathrm{s}^{-1}$
R_p	$25 \times 10^{-6}\ \mathrm{m\ W}^{-1}\ \mathrm{s}^{-1}$
R_a	$-2.5 \times 10^{-6}\ \mathrm{m\ W}^{-1}\ \mathrm{s}^{-1}$

Table S1: Simulation parameters.

References:

[1] Lai, M., Nicholson, J. & Rudolph, W., "Multiple pulse operation of a femtosecond Ti: 315 sapphire laser.", Opt. Commun. 142, 45–49 (1997).

[2] Kitano, H. & Kinoshita, S. Stable multipulse generation from a self-mode-locked Ti: sapphire laser. Opt. Commun. 157, 128–134 (1998).

[3] Kurtz, F., Ropers, C. & Herink, G. Resonant excitation and all-optical switching of femtosecond soliton molecules. Nat. Photonics 14, 9–13 (2020).

[4] Smolorz, S. & Wise, F. Time-resolved nonlinear refraction in femtosecond laser gain media. Opt. Lett. 23, 1381–1383 (1998).

[5] Spielmann C., Curley P. F., Brabec T. & Krausz F. Ultrabroadband femtosecond lasers. IEEE Journal of Quantum Electronics 30 (4), 1100-1114 (1994).

[6] Kalashnikov, V. L., Sorokin, E. & Sorokina, I. T. Multipulse operation and limits of the Kerr-lens mode-locking stability. IEEE J. Quantum Electron. 39, 323–336 (2003).

[7] Salin, F., Piché, M. & Squier, J. Mode locking of Ti: Al 2 O 3 lasers and self-focusing: a Gaussian approximation. Opt. Lett. 16, 1674–1676 (1991).

[8] Cerullo, G., De Silvestri, S. & Magni, V. Self-starting Kerr-lens mode locking of a Ti: sapphire laser. Opt. Lett. 19, 1040–1042 (1994).

[9] Sander M. Y., Birge J., Benedick A., Crespo H. M. & Kärtner F. X. Dynamics of dispersion managed octave-spanning titanium:sapphire lasers. J. Opt. Soc. Am. B **26**, 743-749 (2009)
